# 1,1′,2,2′-Tetra­methyl-3,3′-(*p*-phenyl­enedimethyl­ene)diimidazol-1-ium dibromide

**DOI:** 10.1107/S1600536809026324

**Published:** 2009-07-11

**Authors:** Subramaniam Puvaneswary, Yatimah Alias, Seik Weng Ng

**Affiliations:** a Department of Chemistry, University of Malaya, 50603 Kuala Lumpur, Malaysia

## Abstract

The title imidazolium-based ionic-liquid salt, C_18_H_24_N_4_^2+^·2Br^−^, has the cation lying about a center of inversion. The five-membered imidazole ring is disordered over two positions with the major component having a site occupancy of 0.712 (4); the N-bound methyl substituents are ordered. The imidazole ring is approximately perpendicular to the six-membered phenyl­ene ring [dihedral angle = 80.7 (5)° for the major disorder component and 89.8 (3)° for the other; the two components are off-set by 10.1 (6)°].

## Related literature

For background to imidazolium-based ionic liquid salts, see: Ganesan *et al.* (2008 ▶). For 2,2′-dimethyl-3,3′-(*p*-phenyl­enedimethyl­ene)diimidazol-1-ium dibromide, see: Dobrzańska (2005 ▶). 
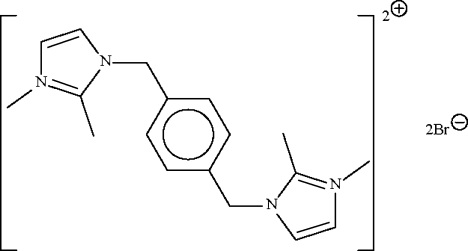


## Experimental

### 

#### Crystal data

C_18_H_24_N_4_^2+^·2Br^−^
*M* *_r_* = 456.23
Monoclinic, 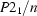

*a* = 8.5689 (4) Å
*b* = 9.8617 (5) Å
*c* = 11.0272 (4) Å
β = 93.222 (3)°
*V* = 930.37 (7) Å^3^
*Z* = 2
Mo *K*α radiation
μ = 4.36 mm^−1^
*T* = 140 K
0.40 × 0.08 × 0.04 mm

#### Data collection

Bruker SMART APEX diffractometer
Absorption correction: multi-scan (*SADABS*; Sheldrick, 1996 ▶) *T*_min_ = 0.274, *T*_max_ = 0.845
5882 measured reflections
2131 independent reflections
1575 reflections with *I* > 2σ(*I*)
*R*_int_ = 0.051

#### Refinement

*R*[*F* ^2^ > 2σ(*F* ^2^)] = 0.039
*wR*(*F* ^2^) = 0.096
*S* = 1.04
2131 reflections
112 parameters
36 restraints
H-atom parameters constrained
Δρ_max_ = 0.54 e Å^−3^
Δρ_min_ = −0.99 e Å^−3^

### 

Data collection: *APEX2* (Bruker, 2008 ▶); cell refinement: *SAINT* (Bruker, 2008 ▶); data reduction: *SAINT*; program(s) used to solve structure: *SHELXS97* (Sheldrick, 2008 ▶); program(s) used to refine structure: *SHELXL97* (Sheldrick, 2008 ▶); molecular graphics: *X-SEED* (Barbour, 2001 ▶); software used to prepare material for publication: *publCIF* (Westrip, 2009 ▶).

## Supplementary Material

Crystal structure: contains datablocks global, I. DOI: 10.1107/S1600536809026324/tk2494sup1.cif

Structure factors: contains datablocks I. DOI: 10.1107/S1600536809026324/tk2494Isup2.hkl

Additional supplementary materials:  crystallographic information; 3D view; checkCIF report

## Figures and Tables

**Fig. 1.**
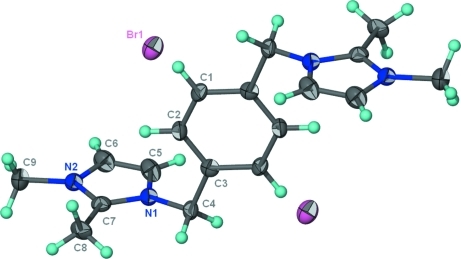
Thermal ellipsoid plot (Barbour, 2001) of [C18H24N4]2+ 2Br- at the 70% probability level; hydrogen atoms are drawn as spheres of arbitrary radius. The imidazolyl ring is disordered; the minor component of the disorder is not shown. The non-H atoms comprising the asymmetric unit are labelled and the unlabelled atoms are related by 1-x, -y, -z.

## supplementary crystallographic information

### Experimental

α,α-Dibromo-*p*-xylene (0.78 g, 3 mmol) and 1,2-dimethylimidazole (0.58 g, 6 mmol) were refluxed in DMF (50 ml) for 3 h. The product that separated from solution was collected and washed with ether. Crystals were grown from its solution in water.

### Refinement

The imidazolyl ring is disordered over two positions (the two N-bound methyl groups are ordered); the major component had a site occupancy = 0.712 (4). The ring was refined as a regular pentagon of 1.35 Å sides. The anisotropic displacement parameters of the primed atoms were restrained to those of the unprimed ones; these were further restrained to be nearly isotropic.

Carbon-bound H-atoms were placed in calculated positions (C—H 0.95 to 0.99 Å) and were included in the refinement in the riding model approximation, with *U*(H) set to 1.2 to 1.5*U*_eq_(C).

### Crystal data

**Table:**

C_18_H_24_N_4_^2+^·2Br^−^	*F*(000) = 460
*M**_r_* = 456.23	*D*_x_ = 1.629 Mg m^−^^3^
Monoclinic, *P*2_1_/*n*	Mo *K*α radiation, λ = 0.71073 Å
Hall symbol: -P 2yn	Cell parameters from 1543 reflections
*a* = 8.5689 (4) Å	θ = 2.8–25.5°
*b* = 9.8617 (5) Å	µ = 4.36 mm^−^^1^
*c* = 11.0272 (4) Å	*T* = 140 K
β = 93.222 (3)°	Prism, colorless
*V* = 930.37 (7) Å^3^	0.40 × 0.08 × 0.04 mm
*Z* = 2	

### Data collection

**Table:**

Bruker SMART APEX diffractometer	2131 independent reflections
Radiation source: fine-focus sealed tube	1575 reflections with *I* > 2σ(*I*)
graphite	*R*_int_ = 0.051
ω scans	θ_max_ = 27.5°, θ_min_ = 2.8°
Absorption correction: multi-scan (*SADABS*; Sheldrick, 1996)	*h* = −11→10
*T*_min_ = 0.274, *T*_max_ = 0.845	*k* = −12→12
5882 measured reflections	*l* = −14→14

### Refinement

**Table:**

Refinement on *F*^2^	Primary atom site location: structure-invariant direct methods
Least-squares matrix: full	Secondary atom site location: difference Fourier map
*R*[*F*^2^ > 2σ(*F*^2^)] = 0.039	Hydrogen site location: inferred from neighbouring sites
*wR*(*F*^2^) = 0.096	H-atom parameters constrained
*S* = 1.04	*w* = 1/[σ^2^(*F*_o_^2^) + (0.0403*P*)^2^ + 0.5004*P*] where *P* = (*F*_o_^2^ + 2*F*_c_^2^)/3
2131 reflections	(Δ/σ)_max_ = 0.001
112 parameters	Δρ_max_ = 0.54 e Å^−^^3^
36 restraints	Δρ_min_ = −0.99 e Å^−^^3^

### Fractional atomic coordinates and isotropic or equivalent isotropic displacement parameters (Å^2^)

**Table:**

	*x*	*y*	*z*	*U*_iso_*/*U*_eq_	Occ. (<1)
Br1	0.46792 (4)	0.59691 (4)	0.76873 (3)	0.02742 (14)	
C1	0.6432 (4)	0.0488 (4)	0.4669 (3)	0.0194 (7)	
H1	0.7414	0.0823	0.4445	0.023*	
C2	0.6148 (4)	0.0331 (3)	0.5895 (3)	0.0194 (7)	
H2	0.6936	0.0558	0.6501	0.023*	
C3	0.4712 (4)	−0.0158 (3)	0.6230 (3)	0.0188 (7)	
C4	0.4343 (4)	−0.0353 (4)	0.7539 (3)	0.0201 (7)	
H4A	0.3204	−0.0246	0.7615	0.024*	0.712 (4)
H4B	0.4631	−0.1287	0.7794	0.024*	0.712 (4)
H4C	0.4003	−0.1303	0.7649	0.024*	0.288 (4)
H4D	0.3452	0.0241	0.7714	0.024*	0.288 (4)
N1	0.5158 (3)	0.0594 (3)	0.8328 (3)	0.0199 (10)	0.712 (4)
C5	0.4760 (4)	0.1910 (3)	0.8444 (3)	0.0259 (11)	0.712 (4)
H5	0.3862	0.2336	0.8072	0.031*	0.712 (4)
C6	0.5862 (4)	0.2513 (2)	0.9182 (3)	0.0270 (13)	0.712 (4)
H6	0.5875	0.3439	0.9421	0.032*	0.712 (4)
N2	0.6941 (3)	0.1570 (3)	0.9523 (3)	0.0207 (9)	0.712 (4)
C7	0.6507 (4)	0.0384 (2)	0.8994 (3)	0.0203 (11)	0.712 (4)
C8	0.7408 (7)	−0.0908 (5)	0.9105 (5)	0.0256 (12)	0.712 (4)
H8A	0.8262	−0.0889	0.8549	0.038*	0.712 (4)
H8B	0.6714	−0.1673	0.8896	0.038*	0.712 (4)
H8C	0.7842	−0.1012	0.9941	0.038*	0.712 (4)
N1'	0.5677 (9)	−0.0057 (8)	0.8456 (6)	0.0199 (10)	0.288
C5'	0.6581 (11)	−0.1058 (6)	0.8950 (8)	0.0259 (11)	0.288
H5'	0.6480	−0.1998	0.8775	0.031*	0.288 (4)
C6'	0.7654 (9)	−0.0488 (8)	0.9736 (8)	0.0270 (13)	0.288
H6'	0.8440	−0.0956	1.0211	0.032*	0.288 (4)
N2'	0.7413 (9)	0.0865 (7)	0.9728 (7)	0.0207 (9)	0.288
C7'	0.6192 (10)	0.1132 (6)	0.8937 (7)	0.0203 (11)	0.288
C8'	0.5509 (19)	0.2515 (14)	0.8644 (14)	0.0256 (12)	0.288
H8'1	0.5473	0.3048	0.9392	0.038*	0.288 (4)
H8'2	0.4448	0.2412	0.8275	0.038*	0.288 (4)
H8'3	0.6162	0.2983	0.8074	0.038*	0.288 (4)
C9	0.8342 (5)	0.1840 (4)	1.0313 (3)	0.0293 (9)	
H9A	0.8278	0.2755	1.0654	0.044*	0.712 (4)
H9B	0.9272	0.1770	0.9840	0.044*	0.712 (4)
H9C	0.8410	0.1175	1.0975	0.044*	0.712 (4)
H9D	0.7798	0.2715	1.0279	0.044*	0.288 (4)
H9E	0.9330	0.1919	0.9912	0.044*	0.288 (4)
H9F	0.8555	0.1577	1.1164	0.044*	0.288 (4)

### Atomic displacement parameters (Å^2^)

**Table:**

	*U*^11^	*U*^22^	*U*^33^	*U*^12^	*U*^13^	*U*^23^
Br1	0.0221 (2)	0.0266 (2)	0.0336 (2)	0.00039 (17)	0.00258 (14)	−0.00512 (16)
C1	0.0122 (19)	0.0253 (17)	0.0208 (17)	−0.0017 (14)	0.0021 (13)	0.0009 (13)
C2	0.016 (2)	0.0235 (18)	0.0183 (17)	0.0002 (14)	−0.0025 (13)	−0.0002 (13)
C3	0.019 (2)	0.0188 (17)	0.0192 (17)	0.0022 (14)	0.0025 (13)	0.0007 (13)
C4	0.013 (2)	0.0275 (18)	0.0194 (17)	−0.0009 (14)	−0.0008 (13)	0.0010 (13)
N1	0.018 (2)	0.025 (2)	0.0168 (18)	−0.0023 (17)	0.0016 (16)	0.0014 (17)
C5	0.025 (3)	0.030 (3)	0.022 (2)	0.008 (2)	0.000 (2)	0.000 (2)
C6	0.027 (3)	0.026 (3)	0.028 (3)	0.006 (2)	0.000 (2)	0.002 (2)
N2	0.020 (3)	0.025 (2)	0.0164 (19)	−0.0032 (18)	0.0019 (16)	0.0007 (17)
C7	0.018 (3)	0.023 (3)	0.021 (2)	0.000 (2)	0.0055 (18)	0.005 (2)
C8	0.021 (3)	0.021 (2)	0.034 (3)	0.009 (2)	0.000 (2)	0.012 (2)
N1'	0.018 (2)	0.025 (2)	0.0168 (18)	−0.0023 (17)	0.0016 (16)	0.0014 (17)
C5'	0.025 (3)	0.030 (3)	0.022 (2)	0.008 (2)	0.000 (2)	0.000 (2)
C6'	0.027 (3)	0.026 (3)	0.028 (3)	0.006 (2)	0.000 (2)	0.002 (2)
N2'	0.020 (3)	0.025 (2)	0.0164 (19)	−0.0032 (18)	0.0019 (16)	0.0007 (17)
C7'	0.018 (3)	0.023 (3)	0.021 (2)	0.000 (2)	0.0055 (18)	0.005 (2)
C8'	0.021 (3)	0.021 (2)	0.034 (3)	0.009 (2)	0.000 (2)	0.012 (2)
C9	0.025 (2)	0.034 (2)	0.027 (2)	−0.0067 (17)	−0.0061 (16)	0.0026 (16)

### Geometric parameters (Å, °)

**Table:**

C1—C3^i^	1.392 (5)	C8—H8A	0.9800
C1—C2	1.396 (4)	C8—H8B	0.9800
C1—H1	0.9500	C8—H8C	0.9800
C2—C3	1.391 (5)	N1'—C5'	1.3500
C2—H2	0.9500	N1'—C7'	1.3500
C3—C1^i^	1.392 (5)	C5'—C6'	1.3500
C3—C4	1.507 (4)	C5'—H5'	0.9500
C4—N1	1.432 (4)	C6'—N2'	1.3500
C4—N1'	1.512 (7)	C6'—H6'	0.9500
C4—H4A	0.9900	N2'—C7'	1.3500
C4—H4B	0.9900	N2'—C9	1.384 (7)
C4—H4C	0.9900	C7'—C8'	1.513 (15)
C4—H4D	0.9900	C8'—H8'1	0.9800
N1—C5	1.3500	C8'—H8'2	0.9800
N1—C7	1.3500	C8'—H8'3	0.9800
C5—C6	1.3500	C9—H9A	0.9800
C5—H5	0.9500	C9—H9B	0.9800
C6—N2	1.3500	C9—H9C	0.9800
C6—H6	0.9500	C9—H9D	0.9800
N2—C7	1.3500	C9—H9E	0.9800
N2—C9	1.468 (4)	C9—H9F	0.9800
C7—C8	1.492 (5)		
			
C3^i^—C1—C2	120.6 (3)	N2—C7—C8	125.2 (3)
C3^i^—C1—H1	119.7	N1—C7—C8	126.8 (3)
C2—C1—H1	119.7	C5'—N1'—C7'	108.0
C3—C2—C1	120.1 (3)	C5'—N1'—C4	121.6 (6)
C3—C2—H2	120.0	C7'—N1'—C4	130.4 (6)
C1—C2—H2	120.0	C6'—C5'—N1'	108.0
C2—C3—C1^i^	119.3 (3)	C6'—C5'—H5'	126.0
C2—C3—C4	122.4 (3)	N1'—C5'—H5'	126.0
C1^i^—C3—C4	118.3 (3)	C5'—C6'—N2'	108.0
N1—C4—C3	112.1 (3)	C5'—C6'—H6'	126.0
C3—C4—N1'	115.1 (4)	N2'—C6'—H6'	126.0
N1—C4—H4A	109.2	C7'—N2'—C6'	108.0
C3—C4—H4A	109.2	C7'—N2'—C9	124.8 (6)
N1'—C4—H4A	129.4	C6'—N2'—C9	126.8 (6)
N1—C4—H4B	109.2	N2'—C7'—N1'	108.0
C3—C4—H4B	109.2	N2'—C7'—C8'	126.2 (8)
H4A—C4—H4B	107.9	N1'—C7'—C8'	125.8 (8)
C3—C4—H4C	108.5	C7'—C8'—H8'1	109.5
N1'—C4—H4C	108.5	C7'—C8'—H8'2	109.5
C3—C4—H4D	108.5	H8'1—C8'—H8'2	109.5
N1'—C4—H4D	108.5	C7'—C8'—H8'3	109.5
H4C—C4—H4D	107.5	H8'1—C8'—H8'3	109.5
C5—N1—C7	108.0	H8'2—C8'—H8'3	109.5
C5—N1—C4	124.6 (3)	N2—C9—H9A	109.5
C7—N1—C4	127.2 (3)	N2—C9—H9B	109.5
N1—C5—C6	108.0	H9A—C9—H9B	109.5
N1—C5—H5	126.0	N2—C9—H9C	109.5
C6—C5—H5	126.0	H9A—C9—H9C	109.5
N2—C6—C5	108.0	H9B—C9—H9C	109.5
N2—C6—H6	126.0	N2'—C9—H9D	109.5
C5—C6—H6	126.0	N2'—C9—H9E	109.5
C6—N2—C7	108.0	H9D—C9—H9E	109.5
C6—N2—C9	124.4 (3)	N2'—C9—H9F	109.5
C7—N2—C9	127.6 (3)	H9D—C9—H9F	109.5
N2—C7—N1	108.0	H9E—C9—H9F	109.5
			
C3^i^—C1—C2—C3	−0.1 (6)	C4—N1—C7—C8	−3.3 (5)
C1—C2—C3—C1^i^	0.1 (6)	N1—C4—N1'—C5'	170.1 (11)
C1—C2—C3—C4	179.8 (3)	C3—C4—N1'—C5'	−98.3 (6)
C2—C3—C4—N1	30.3 (5)	N1—C4—N1'—C7'	−9.2 (5)
C1^i^—C3—C4—N1	−150.0 (3)	C3—C4—N1'—C7'	82.4 (8)
C2—C3—C4—N1'	−3.4 (6)	C7'—N1'—C5'—C6'	0.0
C1^i^—C3—C4—N1'	176.3 (4)	C4—N1'—C5'—C6'	−179.5 (8)
C3—C4—N1—C5	76.5 (4)	N1'—C5'—C6'—N2'	0.0
N1'—C4—N1—C5	178.8 (8)	C5'—C6'—N2'—C7'	0.0
C3—C4—N1—C7	−97.3 (3)	C5'—C6'—N2'—C9	−173.4 (8)
N1'—C4—N1—C7	4.9 (7)	C6'—N2'—C7'—N1'	0.0
C7—N1—C5—C6	0.0	C9—N2'—C7'—N1'	173.5 (8)
C4—N1—C5—C6	−174.8 (3)	C6'—N2'—C7'—C8'	179.7 (11)
N1—C5—C6—N2	0.0	C9—N2'—C7'—C8'	−6.8 (12)
C5—C6—N2—C7	0.0	C5'—N1'—C7'—N2'	0.0
C5—C6—N2—C9	179.0 (3)	C4—N1'—C7'—N2'	179.4 (8)
C6—N2—C7—N1	0.0	C5'—N1'—C7'—C8'	−179.7 (11)
C9—N2—C7—N1	−179.0 (4)	C4—N1'—C7'—C8'	−0.3 (12)
C6—N2—C7—C8	178.0 (4)	C7'—N2'—C9—N2	6.6 (5)
C9—N2—C7—C8	−1.0 (5)	C6'—N2'—C9—N2	179.0 (11)
C5—N1—C7—N2	0.0	C6—N2—C9—N2'	171.4 (8)
C4—N1—C7—N2	174.7 (3)	C7—N2—C9—N2'	−9.8 (7)
C5—N1—C7—C8	−177.9 (4)		

Symmetry codes: (i) −*x*+1, −*y*, −*z*+1.
